# Community size and composition of ammonia oxidizers and denitrifiers in an alluvial intertidal wetland ecosystem

**DOI:** 10.3389/fmicb.2014.00371

**Published:** 2014-07-23

**Authors:** Ziye Hu, Han Meng, Jin-Huan Shi, Nai-Shun Bu, Chang-Ming Fang, Zhe-Xue Quan

**Affiliations:** ^1^Department of Microbiology and Microbial Engineering, School of Life Sciences, Fudan UniversityShanghai, China; ^2^Ministry of Education Key Laboratory for Biodiversity Science and Ecological Engineering, Institute of Biodiversity Science, Fudan UniversityShanghai, China

**Keywords:** community size, community composition, nitrification, denitrification, intertidal wetland

## Abstract

Global nitrogen cycling is mainly mediated by the activity of microorganisms. Nitrogen cycle processes are mediated by functional groups of microorganisms that are affected by constantly changing environmental conditions and substrate availability. In this study, we investigated the temporal and spatial patterns of nitrifier and denitrifier communities in an intertidal wetland. Soil samples were collected over four distinct seasons from three locations with different vegetative cover. Multiple environmental factors and process rates were measured and analyzed together with the community size and composition profiles. We observed that the community size and composition of the nitrifiers and denitrifiers are affected significantly by seasonal factors, while vegetative cover affected the community composition. The seasonal impacts on the community size of ammonia oxidizing archaea (AOA) are much higher than that of ammonia oxidizing bacteria (AOB). The seasonal change was a more important indicator for AOA community composition patterns, while vegetation was more important for the AOB community patterns. The microbial process rates were correlated with both the community size and composition.

## Introduction

The intertidal wetland ecosystem is a type of coastal wetland that is characterized by high primary production rates and intense remineralization in its sediments (Carling, 1982). Intertidal wetlands and estuarine ecosystems are considered the most important land–sea interaction areas in the world. The estuarine intertidal wetland is a natural barrier in purifying territorial pollution and attenuating the riverine load to the sea and plays a key role in controlling marine eutrophication and global nitrogen recycling. Microbial communities in these ecosystems are usually highly abundant and diverse (Wilms et al., 2006).

The Dongtan wetland is an estuarine intertidal wetland near the sea that is located at the eastern end of Chongming Island in the Yangtze River estuary. Chongming Island is the third largest island in China and the largest alluvial island in the world (Gan et al., 2009). Because of its geographic location, organic matter contained in this wetland is carried by both the Yangtze River and the open sea. The Yangtze River carries ~4.68 × 10^8^ tons of sediment to this area annually, and more than half of the sediment accumulates in the estuary region, which contains a large amount of nitrates, ammonium, and organic matter (Chen, 1996) that are primary or secondary substrates for nitrogen transformation processes, including nitrification and denitrification.

Nitrification and denitrification are two principal microbial processes that determine the nitrogen loss from ecosystems, both of which are mainly driven by the activities of microbial communities. Nitrification, including ammonium oxidization and nitrite oxidization, was long believed to be accomplished by a small specific group of bacteria, until the existence of an archaeal ammonium oxidizer was identified about a decade ago (Könneke et al., 2005). Conversely, denitrification has been detected in many different microbial taxa, most of which are phylogenetically distantly related (Zumft, 1992).

In estuaries, the physiological and metabolic features of the aforementioned functional microbial communities in terms of activity, abundance, and composition are constantly changing due to the unstable environmental parameters, such as the C/N ratio (Li et al., 2012; Abell et al., 2013), pH (Cao et al., 2011), temperature (Sahan and Muyzer, 2008; Zheng et al., 2013), and salinity (Mosier and Francis, 2008; Santoro et al., 2008; Jin et al., 2011). Unlike most studies in pelagic or terrestrial systems where the AOA always outnumber the AOB, there are some reports that have shown higher numbers for AOB than AOA in estuaries (Magalhaes et al., 2009; Wankel et al., 2011; Zheng et al., 2014); however, there are also some reports of higher AOA than AOB contents in different estuaries (Beman and Francis, 2006; Li et al., 2012). The actual contribution of AOB and AOA to the ammonium oxidization has also been the subject of intensive debate. There are some reports that showed a correlation of nitrification with AOB (Magalhaes et al., 2009; Wankel et al., 2011), while other reports suggest a high contribution of AOA to nitrification (Caffrey et al., 2007; Jin et al., 2011; Zheng et al., 2014). The relative importance, community structure and abundance of AOB and AOA are complex issues that depend on actual environmental conditions (Berhard and Bollmann, 2010).

Thus, to better understand the *in situ* performance and function of nitrogen cycle microorganisms, including ammonia oxidizers and denitrifiers, dedicated studies of the community size and composition in relation to the environmental conditions and process rates are needed. To this end, we investigated the ammonia-oxidizing and denitrifying communities in the Dongtan wetland ecosystem and examined how the composition and size of these communities correlate with environmental variables, such as season, vegetation types, and corresponding microbial process rates.

## Materials and methods

### Site description and sample collection

**Figure 1:**
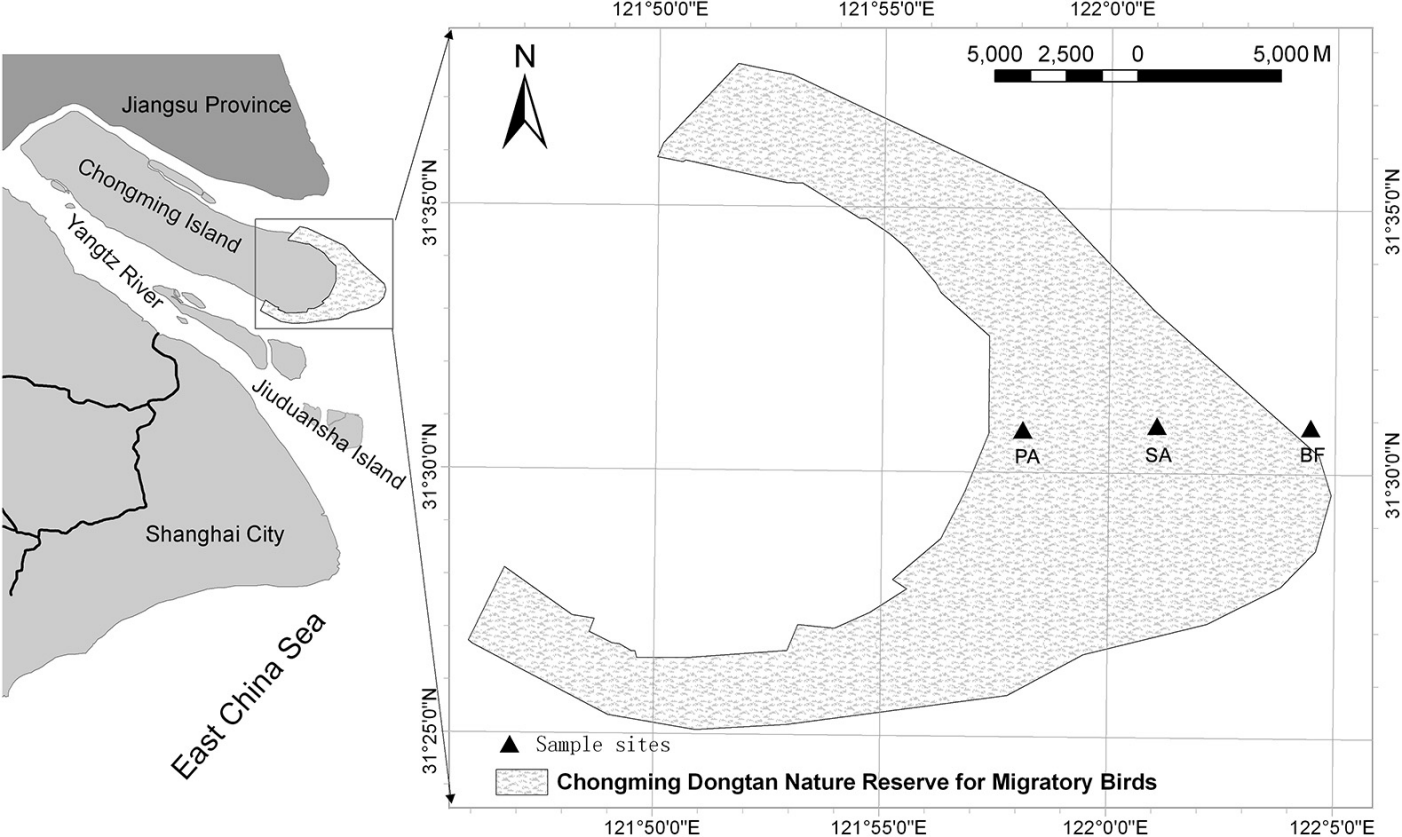
**Map of the study area with sampling sites in the Dongtan wetland, eastern Chongming Island, China**. BF, soil at bare flat (no vegetative cover); SA, soil covered by *Spartina alterniflora*; PA, soil covered by *Phragmites australis*.

The Dongtan wetland lies in the typical subtropical monsoon region of China (Figure 1). The climate in this area is mild and characterized by four distinct seasons. The average annual temperature is 15.3°C, the average summer temperature is 26°C and the average winter temperature is 3°C (Xiao et al., 2009). Vegetation in the sampled site consisted of more than 90 types of vascular plants. Two types were dominant in both the distribution area and total biomass: exotic *Spartina alterniflora* and *Phragmites australis*. The low tidal zone of the Dongtan wetland is mudflats, also known as tidal flats, which have no vegetation cover (bare flat). The middle and high tidal zones are salt marshes. The middle tidal zone is infested with *Spartina alterniflora*, and *Phragmites australis* thrives in the high tidal zone (Zheng et al., 2013).

The samples were collected on 16 July 2008 (summer), 17 October 2008 (autumn), 15 January 2009 (winter), and 17 March 2009 (spring). During this period, the minimum environmental temperature was 6.0°C in January, and the maximum was 34.1°C in July (Table 1). At each sampling, three vegetative types of soil were sampled: soil in a bare flat (BF), soil covered by *Spartina alterniflora* (SA), and soil covered by *Phragmites australis* (PA) (Table 1). For each type of soil, four samples were collected with a cutting ring. Samples were then stored in a cooling box and immediately transported to the laboratory for further analysis. From each sample, 5–20 g of soil was used for measuring the soil's physicochemical properties, and 1–2 g was taken for DNA extraction.

**Table 1:** **The soil properties and microbial process rates for the different seasons and soil types (mean ± SD, *n* = 3)**.

**Season**	**Soil type**	**pH**	**Salinity**	**NH^+^_4_**	**NO^−^_3_**	**Total N**	**Total C**	**C/N ratio**	**Net nitrification rate**	**Potential denitrification rate**	**Temperature^a^**
			**(mg g^−1^ dry soil)**	**(mg N kg^−1^ dry soil)**	**(mg N kg^−1^ dry soil)**	**(g N kg^−1^ dry soil)**	**(g C kg^−1^ dry soil)**		**(μg N g^−1^ dry soil h^−1^)**	**(μg N g^−1^ dry soil h^−1^)**	**(°C)**
Summer	BF	8.09 ± 0.03	2.54 ± 0.04	13.97 ± 1.6	18.42 ± 1.89	0.57 ± 0.22	17.87 ± 4.03	31.31	−17.24 ± 3.06	64.09 ± 6.28	34.1
Summer	SA	7.22 ± 0.11	5.28 ± 0.05	11.91 ± 2.83	12.21 ± 0.26	0.82 ± 0.10	17.46 ± 1.15	21.39	−6.88 ± 3.21	1.21 ± 0.44	34.1
Summer	PA	7.24 ± 0.04	9.00 ± 0.58	8.53 ± 0.61	13.57 ± 1.01	0.79 ± 0.07	17.47 ± 1.16	22.16	−2.98 ± 9.63	24.51 ± 7.04	34.1
Autumn	BF	7.54 ± 0.13	4.60 ± 0.04	13.15 ± 0.49	2.71 ± 0.20	0.94 ± 0.63	20.32 ± 11.47	21.67	121.80 ± 8.11	2.40 ± 1.33	25.8
Autumn	SA	7.33 ± 0.19	7.88 ± 0.16	11.22 ± 0.14	2.99 ± 0.11	0.82 ± 0.21	18.47 ± 2.67	22.5	46.47 ± 3.52	0.48 ± 0.23	25.8
Autumn	PA	9.09 ± 0.34	7.93 ± 0.16	8.61 ± 0.39	2.24 ± 0.55	0.87 ± 0.15	18.52 ± 2.61	21.22	50.19 ± 11.75	1.07 ± 1.08	25.8
Winter	BF	8.89 ± 0.04	8.04 ± 0.09	8.90 ± 0.82	0.27 ± 0.03	0.76 ± 0.34	16.9 ± 3.75	22.21	14.45 ± 2.79	1.72 ± 0.68	6.0
Winter	SA	7.77 ± 0.16	5.70 ± 0.15	8.30 ± 0.26	0.64 ± 0.10	0.72 ± 0.26	16.4 ± 3.15	22.64	10.45 ± 5.58	3.40 ± 2.43	6.0
Winter	PA	8.32 ± 0.14	7.47 ± 0.22	10.61 ± 1.44	0.57 ± 0.09	1.02 ± 0.07	19.8 ± 0.61	19.5	24.49 ± 4.93	3.01 ± 1.22	6.0
Spring	BF	8.59 ± 0.16	7.55 ± 0.02	13.48 ± 2.29	2.87 ± 0.32	0.97 ± 0.53	16.98 ± 3.78	17.54	−3.58 ± 0.74	40.21 ± 8.07	17.2
Spring	SA	7.65 ± 0.04	5.67 ± 0.11	15.44 ± 1.55	2.95 ± 0.30	1.14 ± 0.36	19.92 ± 0.56	17.54	−3.44 ± 2.02	ND^b^	17.2
Spring	PA	7.86 ± 0.06	7.33 ± 0.01	14.59 ± 2.05	3.21 ± 0.33	1.29 ± 0.29	20.17 ± 0.46	15.62	−2.69 ± 1.87	5.69 ± 4.41	17.2

Abbreviations: BF, soil at bare flat; PA, soil covered by Phragmites australis; SA, soil covered by Spartina alterniflora.

a Average temperature value in Shanghai obtained from the historical record at http://www.weatheronline.co.uk.

b Not detected.

### Measurement of the soil properties and microbial process rates

Soil pH and salinities were measured by the HANNA HI 9025 pH/mV/°C waterproof meter (Hanna Instruments, Italy) using an extract of 1:10 dilution of soil with water. The concentrations of nitrate (NO^−^_3_-N) and ammonium (NH^+^_4_-N) were determined according to previously described methods (Solorzano, 1969; Norman et al., 1985). Total carbon (TC) and nitrogen (TN) were determined with a carbon, nitrogen, and sulfur analyzer (CNS-2000, USA) after combustion of the soil samples at 1250°C.

Net nitrification and the potential denitrification rates were used as the microbial process rates in this study. The net nitrification rate was calculated based on the net production of NO^−^_3_-N after incubating fresh soil samples (adjusted to 60% of the water-holding capacity) for 2 weeks aerobically in the dark at 25°C (Verchota et al., 2001; Rutigliano et al., 2009).

The soil denitrification rate was determined as previous described (Yeomans et al., 1992). First, 20 g of fresh soil samples were sealed in glass flasks with butyl rubber stoppers. Ten percent of the atmosphere contained in flasks was then replaced by C_2_H_2_ to prevent reduction of N_2_O. After incubation at 25°C for 48 h, the potential denitrification rates were calculated by measurement of the N_2_O concentration using a gas chromatograph (GC-14B, Shimadzu, Japan).

### DNA extraction and real-time quantitative PCR

DNA was extracted from 0.25 to 1 g aliquots of each sample in triplicate using the UltraClean™ Soil DNA Isolation Kit (Mo-Bio Laboratories, USA) as described before (Li et al., 2012).

Real-time quantitative PCR was performed as described previously (Li et al., 2012), with modification. In short, 10 ng of DNA extract from each sample was used as a template, and the primers and probes are listed in Table 2. The real-time PCR reactions were carried out by an Mx3000P real-time PCR system (Stratagene, USA). The copy number of the 16S rRNA genes of the bacteria and Archaea, the ammonia monooxygenase (*amoA*) genes of the AOB and AOA, as well as the nitrous oxide reductase (*nosZ*) gene of the denitrifiers were determined. To construct standard curves, plasmids containing the target gene were quantified using a NanoDrop 1000 spectrophotometer and were then serially diluted in 10-fold steps before qPCR was performed. Each reaction was performed in triplicate. The results of each reaction are expressed as the copies of each corresponding gene per gram dry-weight of soil.

**Table 2:** **The primers, probes and PCR procedures for real-time quantitative PCR used in this study**.

**Target gene**	**Primer (Probe)**	**Sequence (5′–3′)**	**Thermal profile^a^**	**References**
Bacteria 16S rRNA	1369F	CGGTGAATACGTTCYCGG	1 min at 95°C; 40 cycles of 15 s at 95°C, 1 min at 56°C	Suzuki et al., 2000
	1492R	GGWTACCTTGTTACGACTT		
	(1389F)	CTTGTACACACCGCCCGTC		
Archaea 16S rRNA	ARCH1-1369F	CGGTGAATACGTCCCTGC	1 min at 95°C; 40 cycles of 15 s at 95°C, 1 min at 59°C	Suzuki et al., 2000
	ARCH2-1369F	CGGTGAATATGCCCCTGC		
	1492R	GGWTACCTTGTTACGACTT		
	(1389F)	CTTGTACACACCGCCCGTC		
AOB *amoA*	amoA-1F	GGGGTTTCTACTGGTGGT	1 min at 95°C; 40 cycles of 15 s at 95°C, 30 s at 57°C, 45 s at 72°C	Rotthauwe et al., 1997
	amoA-2R	CCCCTCKGSAAAGCCTTCTTC		
AOA *amoA*	amo-196F	GGWGTKCCRGGRACWGCMAC	1 min at 95°C; 40 cycles of 15 s at 95°C, 40 s at 55°C	Treusch et al., 2005
	amo-277R	CRATGAAGTCRTAHGGRTADCC		
	(amo-247)	CAAACCAWGCWCCYTTKGCDACCCA		
*nosZ*	nosZ2F	CGCRACGGCAASAAGGTSMSSGT	1 min at 95°C; 6 cycles of 15 s at 95°C, 30 s at 65°C with a touchdown of 1°C by cycle, 30 s at 72°C, 15 s at 80°C; 40 cycles of 15 s at 95°C, 15 s at 60°C, 30 s at 72°C and 15 s at 80°C	Henry et al., 2006
	nosZ2R	CAKRTGCAKSGCRTGGCAGAA		

a PCR procedures have been slightly optimized in this study.

### PCR amplification and T-RFLP fingerprinting of the *amoA* and *nosZ* genes

The primer set used for amplification of the AOB *amoA* gene is the same as that for qPCR (Table 2). A partial fragment of the AOA *amoA* gene was amplified using the primer set Arch-amoAF and Arch-amoAR (Francis et al., 2005), and the *nosZ* gene was amplified using the primer set nosZ2F and nosZ2R (Henry et al., 2006). All forward primers were terminally labeled with the reporter dye FAM (6-carboxy-fluorescein). The 50 μl reaction mixture contained 25 μl of PCR MasterMixture (Takara, Japan), 10 pmol of each primer and 20 ng of DNA template. For winter samples, no AOA *amoA* amplicon was obtained. Therefore, a nested PCR was employed to obtain sufficient PCR amplicons. The labeled amplicons were checked by agarose gel electrophoresis and then purified with a Qiagen Gel Extraction Kit (Qiagen, USA). To identify the type of enzymes to be used for T-RFLP fingerprinting and to determine which enzyme showed the highest resolution and least redundancy, an *in silico* computation was performed before digestion. We used *Alu*I, *Hha*I, and *Taq*I for the *amoA* genes of AOB, and *Afa*I, *Hha*I and *Msp*I were used for the *amoA* genes of AOA and the *nosZ* genes of denitrifiers. Each enzyme produced at least six T-RFs in all of the samples. In each T-RFLP assay, terminal fragments (T-RFs) that were not digested with the restriction enzymes were considered redundant and were excluded from the results matrix.

Amplicons were digested with restriction endonucleases as described in the manufacturer's protocol (Takara, Japan). Amplicons of the AOB *amoA* gene were digested with *Alu*I and *Hha*I at 37°C for 3 h and with *Taq*I at 65°C for 3 h, separately. Amplicons of the *amoA* gene of AOA and the *nosZ* gene of the denitrifiers were digested independently with *Hha*I, *Msp*I, and *Afa*I at 37°C for 3 h.

After precipitation with ethanol, DNA fragments were size-separated using an ABI PRISM 3730 Genetic Analyzer (Applied Biosystems). T-RFs from 50 to 600 bp were analyzed with the GeneScan software version 3.7 (Applied Biosystems). The relative abundance of the individual T-RF was calculated as the percentage of total peak area in a given T-RFLP profile, and the T-RFs that contributed less than 5% were not considered for further analysis.

### Statistical analysis

All statistical analyses were carried out using the software packages vegan and BiodiversityR for the program R (R Development Core Team, 2007). Principal component analysis (PCA) was employed to explore by T-RFLP fingerprinting the variations in the community composition. T-RF profiles of the *amoA* genes of ammonia oxidizers and the *nosZ* of denitrifiers were analyzed independently. To determine the factors that best explained the variation in community compositions, redundancy analysis (RDA) was performed. The four different sampling seasons and three different vegetation types (categorical variables) were combined to form the variables season and vegetation, respectively. The results of all of the tests were examined by the permutation test (1000 permutations) at *P* < 0.05. The dissimilarities among all the samples were then calculated using the Bray-Curtis distance, and then, non-metric multidimensional scaling (NMDS) ordination was used to access the similarities of the microbial community composition across different seasons and vegetation types. Soil properties and microbial process rates were concatenated to build a secondary matrix. The vector and surface fitting of these variables within NMDS ordination were performed, and the significance was tested with the permutation test (1000 permutations). The most significant vectors or surfaces were selected to fit against the resulting bi-plots.

For the purpose of exploring the correlations among community size, community composition, environmental variables, and microbial process rates, the values for functional gene copy numbers, T-RFLP fingerprinting profiles, soil properties and nitrification and denitrification rates were transformed into dissimilarity matrices using the Bray-Curtis distance measure. Correspondences between these matrices were evaluated using the Mantel test based on Pearson's product-moment correlation and 1000 permutations. Before analyses, the qPCR data were log_10_ transformed and then divided by each column summation to normalize the numerical distribution and provide variance homogeneity. Data from summer/autumn and from winter/spring were calculated separately during the Mantel test.

## Results

### Soil properties and microbial process rates

The soil pH values of 12 samples varied between 7.2 and 9.1. The salinity changed more dramatically, ranging from 2.54 to 9.00 mg per g of dry soil (Table 1). In the summer and autumn, BF soils had the lowest salinity values compared with the PA and SA. In contrast, BF soil salinity was highest in the winter and spring. This increase was the result of increased aquatic salinity due to the decreased flow rate of the Yangtze River in winter and spring.

The microbial process rates displayed distinct seasonality. The net nitrification rates ranged from −17.24 to 121.80 μg N g^−1^ dry soil h^−1^. All of the samples collected during the summer and spring gave negative net nitrification rates (Table 1), which indicated the denitrifiers had higher activity compared to the nitrifiers in those samples. The net nitrification rates in the autumn and winter were significantly higher than those in the summer and spring (Table 1). In contrast, the potential denitrification rates ranged from 0 to 64.09 μg N g^−1^ dry soil h^−1^. In the summer and spring, the values were generally higher than those in other samples and showed the reverse trend of the nitrification rates. In these two seasons, the potential denitrification rates in BF soil were higher than those in the PA and SA soils (Table 1).

### Functional gene abundance

**Figure 2:**
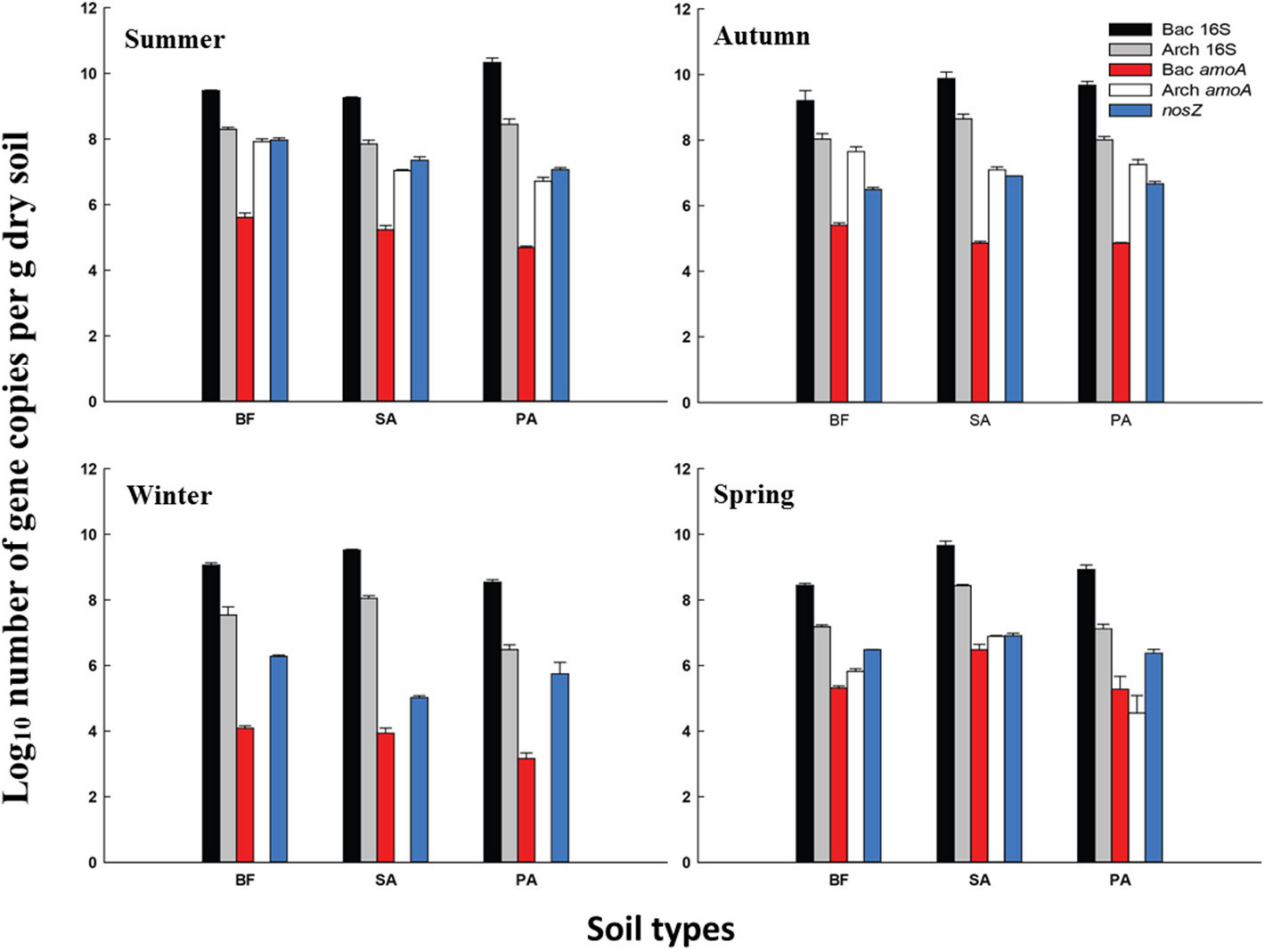
**Average gene copy numbers of 16S rRNA and the functional genes per g of three types of dry soil collected during the four season**. Error bars represent the SD BF, soil at bare flat; SA, soil covered by *Spartina alterniflora*; PA, soil covered by *Phragmites australis*. Bac 16S, Bacterial 16S rRNA gene; Arch 16S, Archaeal 16S rRNA gene; Bac *amoA*, *amoA* gene of AOB; Arch *amoA*, *amoA* gene of AOA; *nosZ*, *nosZ* gene of denitrifier.

The AOB *amoA* gene abundance ranged from 1.5 × 10^3^ to 3.2 × 10^6^ copies g^−1^ dry soil. Three winter samples displayed the lowest values out of the four seasons, which were one to three orders of magnitude lower compared with the others (Figure 2). However, the level of the total bacterial 16S rRNA gene was stable during the study period. In the winter, the copy numbers were not at their lowest but were ~1 order of magnitude lower than the highest level.

The AOA *amoA* genes were always higher in copy number than the AOB *amoA* genes in all of the summer and autumn samples. Interestingly, in the three winter samples, no AOA *amoA* gene was detected. However, there was no significant decrease in the total number of archaeal 16S rRNA gene copies in these samples. To confirm this result, three sampling locations were re-sampled in January 2010 and the qPCR for the AOA *amoA* gene was repeated. These results confirmed the absence of the AOA *amoA* in winter.

In the spring, the AOB *amoA* abundance was similar to the level of the AOA *amoA*, and the difference was less than one order of magnitude. In spring PA, AOB was even higher than the AOA. This was the only AOB dominant sample besides the winter samples (Figure 2). For both the AOB and AOA *amoA* gene abundance, the levels observed in the BF samples were always lowest in spring.

The copy numbers of the *nosZ* genes ranged from 1.0 × 10^5^ to 9.2 × 10^7^ copies g^−1^ of dry soil. Similar to the AOA and AOB *amoA*, the *nosZ* genes also showed seasonal specificity; the lowest level was in winter. Compared with *amoA*, the changes in the level of the *nosZ* gene copy number were relatively small. Within the same season, the levels of gene copy numbers were almost at the same order of magnitude (Figure 2).

### N-cycle community composition revealed by T-RFLP fingerprinting

**Figure 3:**
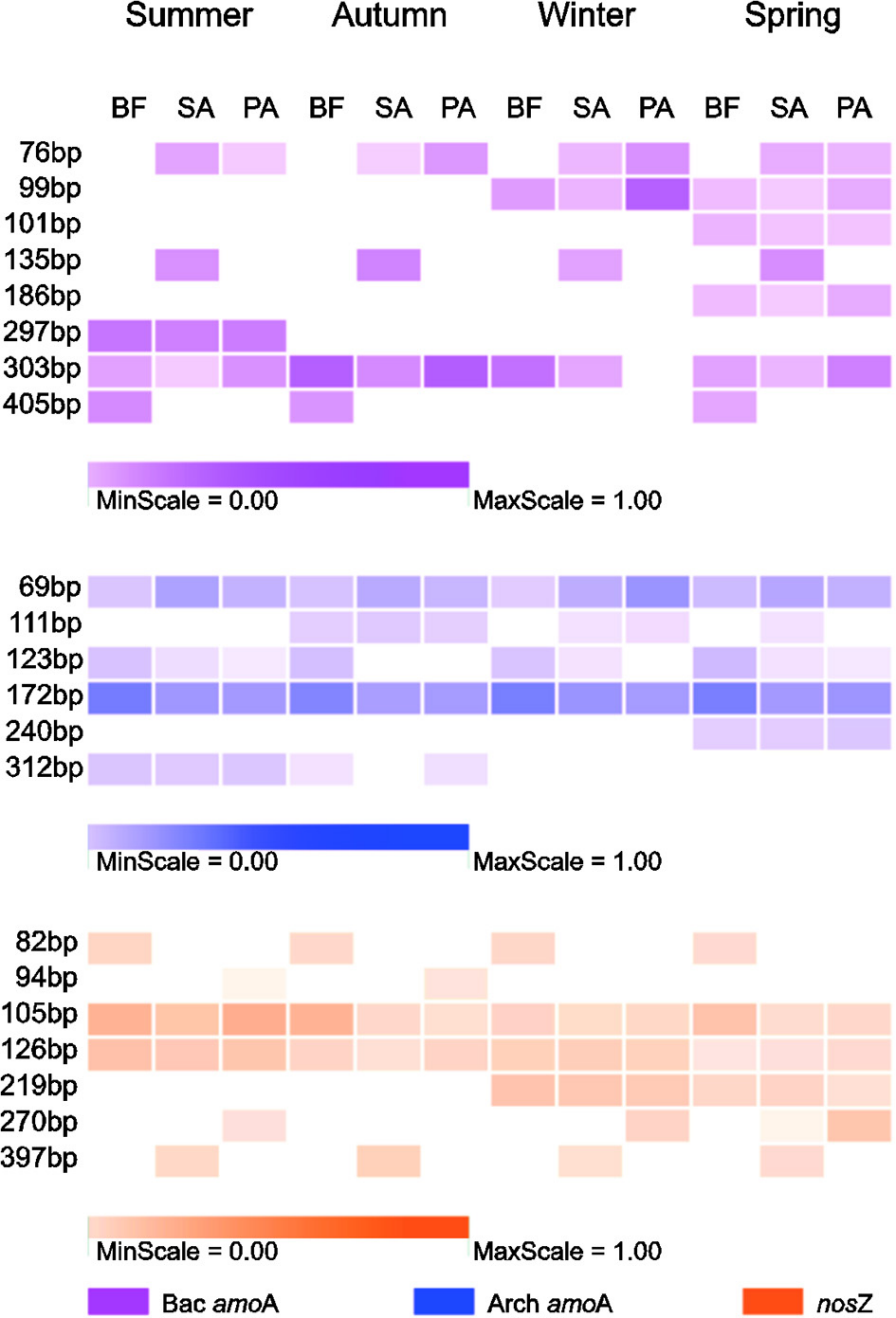
**The community composition revealed by terminal restriction fragment polymorphism (T-RFLP) analysis of the corresponding genes (Bac *amoA*, *amoA* gene of AOB; Arch *amoA*, *amoA* gene of AOA; *nosZ*, *nosZ* gene of denitrifier)**. The colored squares represent relative proportions of terminal restriction fragment (T-RF) during four seasons in three soil types. BF, soil at bare flat; SA, soil covered by *Spartina alterniflora*; PA, soil covered by *Phragmites australis*.

For each gene, we retained the result of the enzyme with the maximum T-RFs and the minimum redundancy. These genes were *Afa*I for the AOA *amoA*, *Taq*I for the AOB *amoA* and *Msp*I for *nosZ*. The most prominent T-RFs, which were present in almost all the samples, were 303 bp in the AOB *amoA*, 69 and 172 bp in the AOA *amoA*, and 105 and 126 bp in *nosZ* (Figure 3).

**Figure 4:**
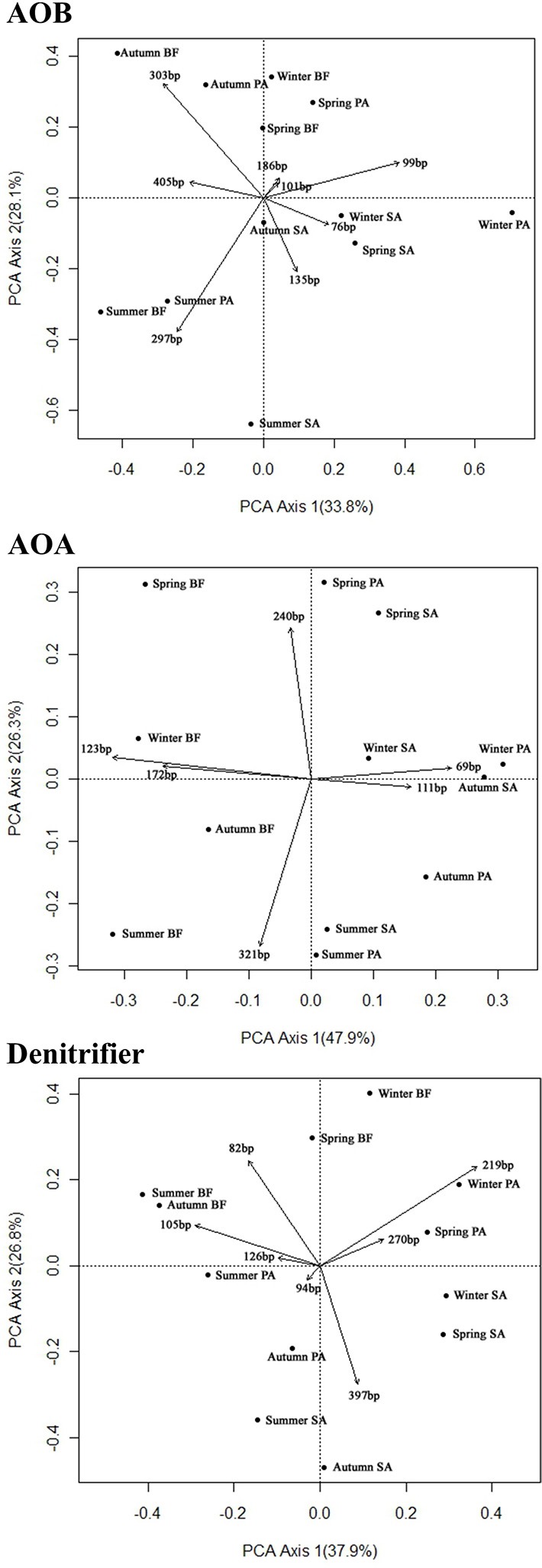
**Principal component analysis (PCA) of the sampling sites based on the composition of the AOB, AOA, and denitrifier communities as determined by the T-RFLP analysis**. Each vector represents an individual T-RF profile. The values in parenthesis indicate the percentage of community variation explained by the axes. BF, soil at bare flat; SA, soil covered by *Spartina alterniflora*; PA, soil covered by *Phragmites australis*.

The T-RF patterns of these nitrogen-cycling communities showed distinct seasonal specificity. For example, the 297-bp T-RF of AOB was only present in the summer samples, and the 186-bp T-RF was only present in the spring samples. Some T-RFs were only detectable in samples from the same or similar soil type (SA and PA); the 76-bp T-RF of the AOB was present in the SA and PA soils but was absent in the BF soils, and the 397-bp T-RF of the denitrifiers was present only in the SA soils in all four seasons. These seasonal and vegetative specificities were further investigated by PCA and RDA analyses of the T-RFLP data (Figure 4). The two PCA axes explained 61.9, 74.2, and 64.7% of the community variation for the AOB, AOA, and denitrifiers, respectively, and clearly separated the three communities of the summer samples from the winter samples. Autumn and spring were also separated, but not as clearly. Generally, the autumn samples shared more similarity with the summer samples, whereas the spring samples were more similar to the winter samples. The clearest separation of vegetation type was between BF and the two other types of soil (SA and PA). However, there was no obvious separation between the SA and PA samples. For the AOB and denitrifiers, the main variations could not be associated with differences in the relative abundance of a single or several T-RFs (Figure 4). For the AOA, the 123- and 69-bp T-RFs were the first two significant factors that determined the main variation of the community (Figure 3). The results of the RDA analysis indicated that all three community compositional variations could be strongly explained by the variables of season and soil type (Table 3).

**Table 3:** **The conditional effects of environmental variables on the community compositional variation as determined by redundant analysis (RDA)**.

**Environmental**	**Constrained proportion^a^**			***F*-ratio**		
**Variable**	**AOB**	**AOA**	**Denitrifier**	**AOB**	**AOA**	**Denitrifier**
pH	0.12	0.06	0.14	1.40 (NS)^b^	0.64 (NS)	1.62 (NS)
Salinity	0.10	0.09	0.08	1.06 (NS)	1.04 (NS)	0.92 (NS)
NH^+^_4_	0.06	0.12	0.03	0.69 (NS)	1.33 (NS)	0.33 (NS)
NO^−^_3_	0.28	0.21	0.21	3.87 (^***^)	2.73 (^*^)	2.72 (^*^)
Total N	0.10	0.16	0.16	1.06 (NS)	1.97 (NS)	1.87 (NS)
Total C	0.11	0.14	0.11	1.26 (NS)	1.67 (NS)	1.22 (NS)
C/N ratio	0.12	0.07	0.07	1.41 (NS)	0.81 (NS)	0.81 (NS)
NiR	0.13	0.11	0.04	1.55 (NS)	1.25 (NS)	0.41 (NS)
DNiR	0.19	0.21	0.16	2.30 (^*^)	2.68 (^*^)	1.93 (NS)
Season	0.51	0.49	0.44	2.83 (^***^)	2.54 (^*^)	2.10 (^*^)
Vegetation type	0.33	0.41	0.46	2.22 (^*^)	3.15 (^*^)	3.85 (^***^)

Abbreviations: NiR, net nitrification rates; DNiR, potential denitrification rates.

a Constrained proportion represents the proportion of each variable explained by the RDA.

b NS, not significant at P > 0.05;

* 0.01 < P ≤ 0.05;

*** P ≤ 0.001.

### Statistical analyses

**Figure 5:**
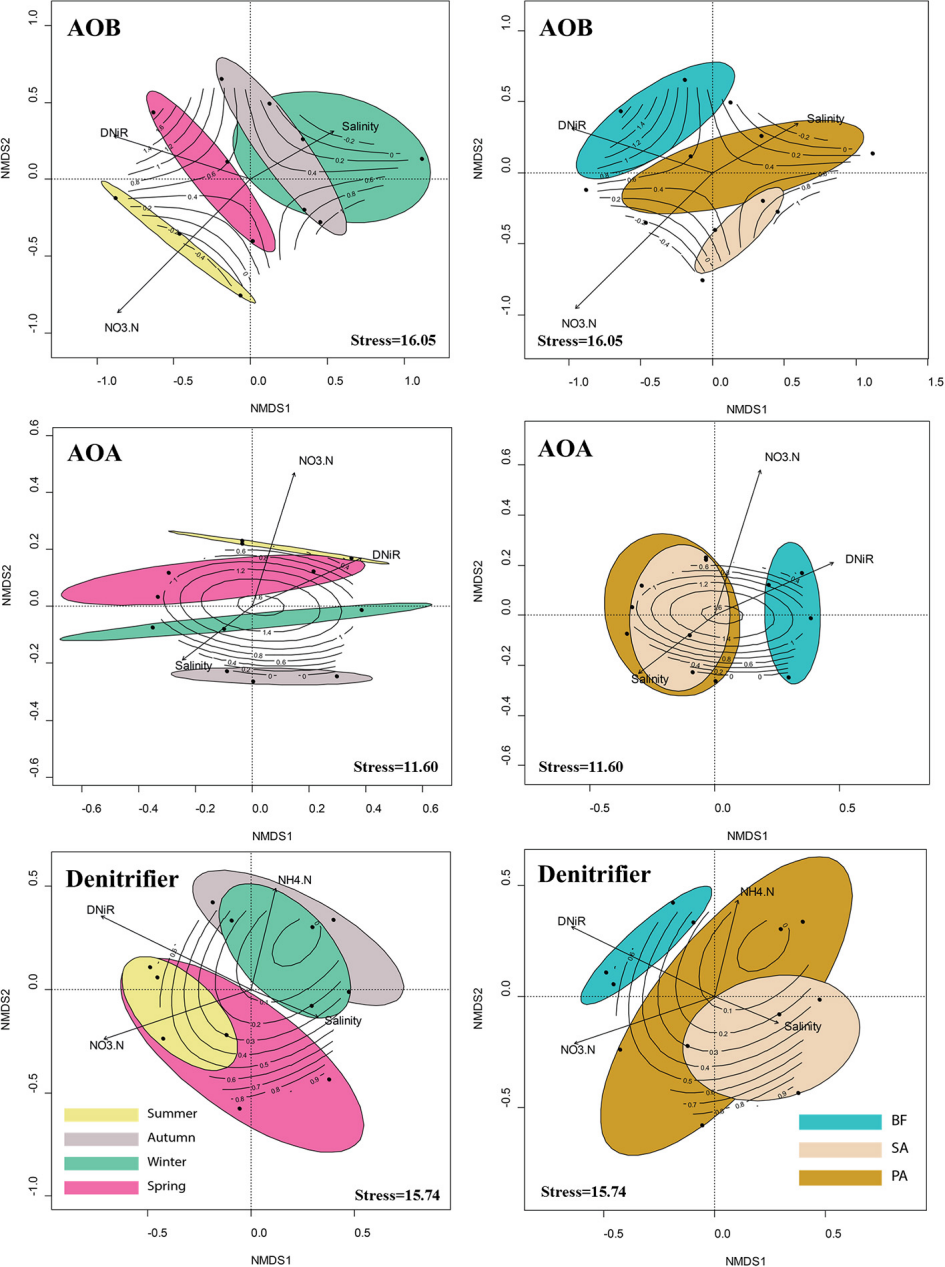
**Non-metric multidimensional scaling (NMDS) ordination of sampling sites based on the composition of the AOB, AOA, and denitrifier communities as determined by the T-RFLP analysis**. The seasonal and vegetative patterns of the communities are visualized by enclosing the samples from the same season or same soil type in a colored ellipse (at 95% confidence). Significant environmental variables (*P* < 0.05) were bi-plotted as vectors. The lengths of the vectors indicate the strength of the correlation between the variables and the ordination scores. Each vector points to the direction of increase for a given variable. Contours represent the net nitrification rate data fit to ordination scores. BF, soil at bare flat; SA, soil covered by *Spartina alterniflora*; PA, soil covered by *Phragmites australis*.

NMDS analysis gave clear separation of the community size/composition in the winter/spring and in the summer/autumn (Figure 5). Soil sample in the summer and autumn shared a greater number of similar environmental factors, such as temperature and substrate concentrations, than the same samples collected in the winter and spring. To find more potential relationships between community size/composition and environmental factors, we combined two seasons together and performed the mantel test separately, and the correlations are listed in Tables 4, 5.

**Table 4:** **Mantel correlations and their significance for the environmental variables, community size and community composition in the summer and autumn**.

	**pH**	**Salinity**	**NH^+^_4_**	**NO_3_^−^**	**Total N**	**Total C**	**C/N ratio**	**NiR**	**DNiR**	**Bac 16S**	**Arch 16S**	**Bac *amoA***	**Arch *amoA***	***nosZ***	**AOB T-RFLP**	**AOA T-RFLP**	***nosZ* T-RFLP**
**SOIL PARAMETERS AND MICROBIAL PROCESS RATES**																	
pH	–	NS	NS	^*^	NS	NS	NS	NS	NS	NS	NS	NS	NS	NS	^*^	NS	NS
Salinity	0.04	–	^***^	^*^	NS	^*^	^***^	^***^	^***^	NS	NS	^***^	^**^	^***^	^***^	^***^	^**^
NH^+^_4_	0.09	**0.45**	–	NS	NS	NS	NS	NS	NS	NS	NS	^***^	^**^	NS	NS	^*^	^*^
NO^−^_3_	**0.18**	**0.18**	−0.01	–	NS	NS	NS	^***^	^**^	^***^	NS	NS	^*^	^***^	^***^	^***^	^*^
Total N	−0.16	−0.01	−0.05	−0.02	–	^***^	^**^	NS	NS	^**^	NS	NS	NS	NS	^*^	NS	NS
Total C	−0.07	**0.23**	0.14	0.10	**0.52**	–	^*^	^*^	NS	^**^	NS	NS	NS	^*^	^*^	NS	NS
C/N ratio	−0.06	**0.58**	0.07	0.05	**0.38**	**0.17**	–	^**^	^**^	NS	NS	^***^	^***^	^**^	^***^	^***^	^**^
NiR	0.00	**0.52**	0.10	**0.72**	0.07	**0.22**	**0.37**	–	^**^	NS	NS	^***^	^***^	^*^	^***^	^***^	NS
DNiR	−0.01	**0.42**	0.06	**0.39**	0.02	0.10	**0.29**	**0.36**	–	NS	NS	^**^	^**^	^**^	NS	NS	^***^
**COMMUNITY SIZE**																	
Bac 16S	0.05	0.07	0.03	**0.79**	**0.26**	**0.31**	0.07	0.07	0.05	–	^**^	^*^	^**^	^***^	^***^	^***^	^***^
Arch 16S	0.16	0.02	0.03	0.06	−0.11	0.15	−0.14	−0.06	0.16	**0.28**	–	NS	NS	NS	NS	NS	NS
Bac *amoA*	−0.03	**0.84**	**0.52**	0.06	0.07	0.09	**0.41**	**0.41**	**0.29**	**0.16**	0.01	–	^***^	^**^	^***^	^**^	^**^
Arch *amoA*	0.07	**0.63**	**0.32**	**0.17**	0.14	0.15	**0.56**	**0.42**	**0.35**	**0.28**	−0.01	**0.58**	–	^*^	^***^	^***^	^***^
*nosZ*	0.02	**0.71**	0.19	**0.61**	**0.14**	**0.45**	**0.49**	**0.28**	**0.42**	**0.56**	0.08	**0.48**	**0.15**	–	^***^	^***^	^***^
**COMMUNITY COMPOSITION**																	
AOB T-RFLP	**0.20**	**0.34**	0.15	**0.64**	**0.17**	**0.12**	**0.42**	**0.58**	0.05	**0.71**	0.15	**0.52**	**0.47**	**0.37**	–	^***^	^***^
AOA T-RFLP	0.08	**0.55**	**0.22**	**0.65**	0.13	0.02	**0.44**	**0.65**	0.02	**0.58**	−0.02	**0.31**	**0.49**	**0.68**	**0.84**	–	^***^
*nosZ* T-RFLP	0.16	**0.29**	**0.27**	**0.27**	0.13	0.01	**0.33**	0.08	**0.38**	**0.41**	0.11	**0.36**	**0.40**	**0.71**	**0.71**	**0.72**	–

Abbreviations: NiR, net nitrification rates; DNiR, potential denitrification rates; Bac 16S, Bacterial 16S rRNA gene; Arch 16S, Archaeal 16S rRNA gene; Bac amoA, amoA gene of AOB; Arch amoA, amoA gene of AOA; nosZ, nosZ gene of denitrifier. NS, not significant at P > 0.05;

*** *P* ≤ 0.001;

** 0.001 < P ≤ 0.01;

* 0.01 < P ≤ 0.05. Bold values indicate a significance of <0.05.

**Table 5:** **Mantel correlations and their significance for environmental variables, community size and community composition in winter and spring**.

	**pH**	**Salinity**	**NH^+^_4_**	**NO^−^_3_**	**Total N**	**Total C**	**C/N Ratio**	**NiR**	**DNiR**	**Bac 16S**	**Arch 16S**	**Bac *amoA***	**Arch *amoA***	***nosZ***	**AOB T-RFLP**	**AOA T-RFLP**	***nosZ* T-RFLP**
**SOIL PARAMETERS AND MICROBIAL PROCESS RATES**																	
pH	–	^***^	NS	^*^	^***^	^***^	^***^	NS	NS	NS	^*^	NS	NS	NS	^**^	^***^	^***^
Salinity	**0.54**	–	NS	NS	NS	NS	NS	NS	NS	NS	^***^	^*^	NS	^**^	NS	NS	^***^
NH^+^_4_	0.07	0.06	–	^***^	NS	NS	NS	^***^	^*^	NS	NS	^**^	^***^	^**^	NS	NS	^*^
NO^−^_3_	**0.19**	0.03	**0.67**	–	NS	NS	^*^	^***^	NS	^**^	NS	^***^	^***^	^***^	^***^	^***^	^**^
Total N	**0.69**	0.16	0.02	0.13	–	^**^	^***^	NS	NS	NS	NS	NS	NS	NS	NS	NS	NS
Total C	**0.65**	0.15	−0.08	0.09	**0.55**	–	^***^	NS	NS	NS	NS	NS	NS	NS	NS	NS	NS
C/N Ratio	**0.68**	0.14	0.15	**0.24**	**0.64**	**0.80**	–	NS	NS	NS	NS	NS	NS	NS	NS	NS	NS
NiR	0.02	−0.04	**0.58**	**0.84**	−0.05	−0.05	0.03	–	^**^	^*^	^*^	^*^	^**^	NS	^***^	NS	NS
DNiR	0.07	0.19	**0.18**	0.07	0.04	0.09	0.05	0.20	–	NS	NS	^***^	^***^	NS	NS	NS	^**^
**COMMUNITY SIZE**																	
Bac 16S	0.01	0.03	0.13	**0.32**	0.11	0.10	0.14	**0.26**	0.09	–	^**^	^***^	^***^	^**^	^*^	^*^	^*^
Arch 16S	**0.26**	**0.73**	0.04	0.08	0.10	0.15	0.03	**0.22**	0.08	**0.42**	–	^***^	NS	NS	^***^	^***^	^**^
Bac *amoA*	0.03	**0.16**	**0.54**	**0.60**	0.02	0.02	0.08	**0.28**	**0.42**	**0.55**	**0.39**	–	^***^	^***^	^***^	^***^	^***^
Arch *amoA*	0.02	0.09	**0.60**	**0.71**	0.03	−0.01	0.13	**0.35**	**0.51**	**0.43**	0.12	**0.70**	–	NS	^**^	^**^	^***^
*nosZ*	0.09	**0.47**	**0.29**	**0.36**	−0.02	0.03	−0.02	0.17	0.13	**0.56**	0.08	**0.43**	0.19	–	^***^	^***^	^*^
**COMMUNITY COMPOSITION**																	
AOB T-RFLP	**0.37**	0.13	0.18	**0.49**	0.06	0.15	0.11	**0.53**	0.13	**0.21**	**0.43**	**0.60**	**0.36**	**0.48**	–	^**^	^**^
AOA T-RFLP	**0.53**	0.17	0.17	**0.52**	0.01	0.12	0.15	0.11	−0.04	**0.24**	**0.61**	**0.46**	**0.28**	**0.35**	**0.53**	–	^***^
*nosZ* T-RFLP	**0.51**	**0.45**	**0.24**	**0.28**	0.09	0.06	0.16	0.03	**0.30**	**0.38**	**0.44**	**0.42**	**0.44**	**0.30**	**0.56**	**0.60**	–

Abbreviations: NiR, net nitrification rates; DNiR, potential denitrification rates; Bac 16S, Bacterial 16S rRNA gene; Arch 16S, Archaeal 16S rRNA gene; Bac amoA, amoA gene of AOB; Arch amoA, amoA gene of AOA; nosZ, nosZ gene of denitrifier. NS, not significant at P > 0.05;

*** *P* ≤ 0.001;

** 0.001 < P ≤ 0.01;

* 0.01 < P ≤ 0.05. Bold values indicate a significance of <0.05.

Our statistical analyses, however, did not find any significant correlation between pH and community size in any season (Tables 4, 5). This result was not surprising because the three vegetative types of soils we studied had similar pH values (Table 1). Our data showed a significant correlation between the differences in the salinity and community size and composition in the summer and autumn samples. In the winter and spring samples, only the difference in the AOA community composition correlated significantly with the differences in the salinity. Other soil properties, such as the C/N ratios, significantly correlated with the AOB, AOA, and denitrifier community sizes and compositions in the summer and autumn. However, similar to salinity, these correlations were no longer significant in the winter and spring.

## Discussion

In many environments, microbial communities display both spatial and temporal patterns that may be associated with changes in the community size and composition. In this study, over four seasons, we sampled soil covered by three different types of vegetation (including no vegetative cover) and analyzed which factor affects the community most strongly.

Many previous studies have demonstrated a greater abundance of archaeal than bacterial *amoA* genes in many environments (Leininger et al., 2006; He et al., 2007; Lam et al., 2007; Wuchter et al., 2007; Nicol et al., 2008; Hong et al., 2013), including estuaries (Beman and Francis, 2006; Abell et al., 2010; Bernhard et al., 2010; Li et al., 2012). Our data from the summer and autumn samples taken from locations with different vegetative cover showed that the AOA were dominant; however, the pattern was not the same in the winter and spring samples (Figure 2). In the winter, the AOA *amoA* gene was completely undetected, while the AOB *amoA* gene copy numbers also declined but not as dramatically as for the AOA (Figure 2). In March 2007, the AOA were also undetectable in the bare flat soil of the Dongtan wetland, the same site used in this study (Li et al., 2012). A slight decrease in the AOA abundance in the winter has been previously reported in a study of long-term fertilized red soil (He et al., 2007). The differences in the AOA community size were significantly correlated with differences in the net nitrification rates in the summer and autumn (Tables 4, 5). In the spring, although the AOA displayed relatively high *amoA* gene abundance, the net nitrification rate still decreased (Table 1 and Figure 2). This result suggests that in the winter and spring, the nitrification may be principally affected by the AOB instead of the AOA. The seasonal variation in the SE USA coastal waters showed that the AOA were 100 to 1000-fold more abundant during their peak than at other times of the year, whereas the abundance of AOB varied <10-fold over the same period (Hollibaugh et al., 2014). Although year-round AOA domination in subtropical macrotidal estuary sediment in Australia has been recently reported in which the AOA community's abundance during the four seasons displayed no significant changes (Abell et al., 2010), this may be related to the less dramatic seasonal differences in temperature during the annual cycle in the Australian system (19–30°C) vs. Shanghai (6.0–34.1°C). A previous study indicated that temperature is one of the most important factors controlling the distribution of both the AOB and AOA (Sahan and Muyzer, 2008). In combination with these previous findings, our results confirmed that temperature may be the primary seasonal factor affecting ammonia oxidizer-community size, and the seasonal impacts on the AOA-community size are much greater than the AOB.

The denitrifier community size, estimated by *nosZ* gene abundance, also displayed clear seasonal specificity with the lowest level observed in the winter. In the summer and autumn, a significant correlation between the potential denitrification rates and denitrifier community size was observed. These findings are consistent with a recent study that showed denitrifier community size was related to the corresponding process rate (Patra et al., 2006). However, in our study, this correlation was not significant in the winter and spring. As was the case with ammonia oxidizers, vegetation had a negligible effect on the denitrifier community size within this narrow range of soils. Because the *nosZ* gene primer set could not cover atypical *nosZ* genes (Orellana et al., 2014) and incomplete denitrifiers do not contain the *nosZ* gene (Henry et al., 2006), the denitrifiers detected in this study were only the complete denitrifiers that can convert nitrate to nitrogen gas.

Statistical analysis of the T-RFLP fingerprinting results (Figure 5) indicated that seasonal change was the most important indicator for the AOA community composition patterns in this wetland ecosystem, and vegetation was more important for the AOB community patterns. This finding is in agreement with previous studies that suggested vegetation has different effects on different microbial communities (Patra et al., 2006; Wang et al., 2013). The Mantel test results indicate that the functional gene abundances significantly correlated with the T-RFLP fingerprinting results, both in the summer/autumn and in the winter/spring (Tables 4, 5), thus implying that there was seasonality in the growth or death of the community members.

Interestingly, in all seasons, the differences in the AOB community size and composition were significantly correlated with the differences in the net nitrification rates; and the differences in the denitrifier community composition were significantly correlated with the differences in the potential denitrification rates. Studies in other soil environments indicated that community composition plays a minor role for the process rates when compared with the role of community size (Hallin et al., 2009). However, our results suggest that the community size as well as the community composition affects the microbial process rates in the environment.

In this study, distinct changes in the AOB-, AOA-, and denitrifier-community sizes and compositions in during all four seasons and three vegetative types of soils were found. All of the community sizes showed strong seasonal specificities. The changes in the community composition showed distinct correlations based on both the season and vegetation type. Specifically, the composition of the AOA community was more significantly correlated with seasonal factors and the denitrifier- community composition was more significantly correlated with vegetation type. A unique absence of AOA in winter was observed, which suggests that seasonal factors have the most significant effects on this community. Both the community size and community composition contributed to the changes in the process rates, including the nitrification and denitrification rates. However, this study only included limited sample locations and has been performed within a time frame of only 1 year. To understand the issue extensively, replicate site locations with the analogous vegetative covers should be studies over several seasonal cycles.

### Conflict of interest statement

The authors declare that the research was conducted in the absence of any commercial or financial relationships that could be construed as a potential conflict of interest.
